# Development of the SciRAP Approach for Evaluating the Reliability and Relevance of *in vitro* Toxicity Data

**DOI:** 10.3389/ftox.2021.746430

**Published:** 2021-10-15

**Authors:** Nicolas Roth, Johanna Zilliacus, Anna Beronius

**Affiliations:** ^1^ Swiss Centre for Applied Human Toxicology (SCAHT), University of Basel, Basel, Switzerland; ^2^ Department of Pharmaceutical Sciences, University of Basel, Basel, Switzerland; ^3^ Institute of Environmental Medicine, Karolinska Institutet, Solna, Sweden

**Keywords:** SciRAP, reliability, relevance, *in vitro* data, data quality, health risk assessment (HRA)

## Abstract

Efficient and successful integration of data generated from non-animal test methods must rely on reliable and relevant data. It is important therefore to develop tools and criteria that facilitate scientifically sound, structured, and transparent evaluation of reliability and relevance of *in vitro* toxicity data to efficiently inform regulatory hazard and risk assessment. The *Science in Risk Assessment and Policy* (SciRAP) initiative aims to promote such overarching goals. We present the work to develop and refine the SciRAP tool for evaluation of reliability and relevance of *in vitro* studies for incorporation on the SciRAP web-based platform (www.scirap.org). In the SciRAP approach, reliability evaluation is based on criteria for reporting quality and methodological quality, and is explicitly separated from relevance evaluation. The SciRAP *in vitro* tool (version 1.0) was tested and evaluated during an expert test round (April 2019-September 2020) on three *in vitro* studies by thirty-one experts from regulatory authorities, industry and academia from different geographical areas and with various degree of experience in *in vitro* research and/or human health risk assessment. In addition, the experts answered an online survey to collect their feedback about the general features and desired characteristics of the tool for further refinement. The SciRAP *in vitro* tool (version 2.0) was revised based on the outcome of the expert test round (study evaluation and online survey) and consists of 24 criteria for evaluating “*reporting quality*” (reliability), 16 criteria for “*methodological quality*” (reliability)*,* and 4 items for evaluating relevance of *in vitro* studies. Participants were generally positive about the adequacy, flexibility, and user-friendliness of the tool. The expert test round outlined the need to (i) revise the formulation of certain criteria; (ii) provide new or revised accompanying guidance for reporting quality and methodological quality criteria in the “*test compounds and controls*,” “*test system*,” and “*data collection and analysis*” domains; and (iii) provide revised guidance for relevance items, as general measures to reduce inter-expert variability. The SciRAP *in vitro* tool allows for a structured and transparent evaluation of *in vitro* studies for use in regulatory hazard and risk assessment of chemicals.

## Introduction

The evaluation of reliability and relevance of individual toxicity studies is a key step as part of the weight of evidence when conducting hazard and risk assessment of chemicals. Structured approaches to reliability and relevance evaluation, based on predefined assessment criteria, are needed for reaching evidence-based conclusions on chemical hazards or risks. This contributes to reducing associated uncertainties in the assessment, adequately inform risk management, and maintain credibility and public trust in the risk analysis process. When concluding on potential hazards or risks of chemicals, risk assessors have to evaluate, synthesize, and integrate data from a number of evidence streams (e.g., *in vitro*, *in silico*, *in vivo*, epidemiological). In the regulatory setting, studies conducted in accordance with standardized test guidelines (e.g., OECD Test Guidelines) are often considered reliable by default, whereas non-standard studies generally have to undergo a more thorough evaluation. Several methods have been proposed for evaluating the reliability/validity/quality of *in vivo* animal (Klimisch et al., 1997; ECETOC, 2009; Schneider et al., 2009; Yang et al., 2013; Maxim and van de Sluijs 2014; Kaltenhäuser et al., 2017; Fernández-Cruz et al., 2018) and human (Money et al., 2013) toxicity studies. However, generic methods for evaluating the reliability of *in vitro* data are more limited (Klimisch et al., 1997; Schneider et al., 2009), with some tools focusing on specific application contexts such as pesticides (Kaltenhäuser et al., 2017) and nanomaterials (Fernández-Cruz et al., 2018) (for a review, see Roth and Ciffroy, 2016).

Many *in vitro* tests that are relevant for use in hazard and risk assessment lack standardised test guidelines. A lot of efforts are currently invested in developing good *in vitro* reporting standards (e.g., CAAT Good *In Vitro* Reporting Standards initiative (GIVReST), Hartung et al., 2019). The paradigm shift in toxicity testing (NRC, 2007) towards the use of alternative non-animal methods and the development of systems toxicology approaches (Smirnova et al., 2018), has led to a substantial increase in available *in vitro* and mechanistic data for regulatory risk assessment (Hartung, 2010). Efficient and successful integration of New Approach Methodologies (NAMs) data for use in Next Generation Hazard and Risk Assessment (NGRA) must then rely on reliable and relevant data. It is important therefore to develop structured tools and criteria that facilitate scientifically sound, systematic, and transparent evaluation of reliability and relevance of *in vitro* toxicity data to efficiently inform decision-making.

The Science in Risk Assessment and Policy (SciRAP) initiative aims to promote structured and transparent evaluation of toxicity data for chemical risk assessment, and bridge the gap between academic research and chemicals regulation and policy (Molander et al., 2015; Beronius et al., 2018). Criteria and tools for the evaluation of reliability and relevance of animal *in vivo*, *in vitro* and ecotoxicity data have been developed and are publicly available on the web-based SciRAP platform (www.scirap.org). The SciRAP platform includes the Criteria for Reporting and evaluating ecotoxicity Data (CRED) (Moermond et al., 2016), which are promoted in several EU guidance documents and in the EU Water Framework Directive. The SciRAP *in vivo* tool was first published on the platform in 2014 and has been since further developed based on user’s feedback (Beronius et al., 2014, 2018).

The SciRAP approach clearly separates reliability from relevance evaluation. Reliability evaluation of *in vitro* and *in vivo* toxicity data is further separated into an evaluation of the study’s “*reporting quality*” (RQ) and “*methodological quality*” (MQ). Reliability “*criteria*” are also distinguished from relevance “*items*.” Reliability criteria correspond to standards against which particular elements and aspects of a study need to be judged, which are considered to reflect intrinsic/inherent properties of the data, system or method that are independent from the context of application under consideration. Criteria cover specific aspects of the test compounds and controls, the test system, the administration of the test compound, and the data collection and analysis. For RQ, there are also criteria addressing the disclosure of funding and competing interests. The SciRAP reliability criteria are primarily based on requirements and recommendations in relevant OECD Test Guidelines (see section on *Expert Test Round* below). In contrast, the relevance items have to be interpreted in the specific context of the risk assessment and the question to be answered (problem formulation), and relate to the extent to which data and tests are appropriate (fit-for-purpose) for their intended use, which are independent from their intrinsic quality. Each reliability criterion may be judged as “*fulfilled*,” “*partially fulfilled*,” “*not fulfilled*,” or “*not determined*,” whereas each relevance item may be judged as “*relevant*,” “*indirectly relevant*,” “*not relevant*,” or “*not determined*,” by choosing from a drop-down menu in the online tool. It is also possible to provide comments for each criterion and item. SciRAP users can generate their own study evaluation report by exporting the results as an Excel file. Each SciRAP report summarises ratings for all reliability criteria and relevance items, and provides colour profiles in the form of: (i) bar diagrams for RQ and MQ criteria; and (ii) pie charts for the relevance items.

The aim of the work presented here was to develop and refine the SciRAP tool for evaluation of reliability and relevance of *in vitro* studies, to be incorporated on the SciRAP web-based platform. It should be noted that the purpose was not to conduct validation of the tool for evaluating *in vitro* data neither to quantitatively assess e.g., the inter-rater or intra-rater reliability, nor the performance of the tool.

## Methods

### Development of SciRAP *in vitro* Tool Version 1.0

The SciRAP approach for evaluating the reliability and relevance of *in vivo* studies (Beronius et al., 2018) was used as the basis for developing a similar evaluation approach for *in vitro* studies, including criteria for evaluating RQ and MQ, as well as items to consider in the evaluation of relevance. In order to formulate specific criteria and items, requirements and recommendations for designing and performing *in vitro* studies stated in relevant OECD Test Guidelines were reviewed, including but not limited to the human *in vitro* skin sensitization assays (OECD, 2016a), the Estrogen Receptor Agonists and Antagonists assays (OECD, 2016b), and the Fish Embryo Acute Toxicity Test (OECD, 2013). The OECD Test Guidelines were specifically scrutinized in terms of requirements and recommendations concerning:

• The test system
• Administration of test compound
• Choice of methods for measuring the intended endpoints
• Observations and measurements
• Reporting

In addition, the OECD Guidance Document No. 211 for Describing Non-Guideline *In Vitro* Test Methods (OECD, 2017) and the OECD Guidance Document No 286 on Good *In Vitro* Method Practices (GIVIMP) (OECD, 2018) were reviewed for additional recommendations that could provide basis for developing the reliability criteria and relevance items, as well as for guidance provided within the SciRAP tool. Guidance was initially developed to facilitate the evaluation of MQ and relevance, as well as to improve consistency between evaluators. The criteria/items and guidance were then incorporated into the SciRAP web-based platform (www.scirap.org) for *in vitro* data evaluation (tool version 1.0).

### Expert Test Round

#### Expert Test Round Procedure

The SciRAP *in vitro* tool version 1.0 was tested and evaluated during an expert test round conducted from April 2019 to September 2020. The aim of the test round was to assess the practical use of the SciRAP *in vitro* approach as a whole among intended end users from different sectors and geographical areas.

Participation in the expert test round included two assignments, first to evaluate three studies using the SciRAP tool and second, to complete an online survey (the questions participants had to answer are provided as Supplementary Table S1). The purpose of the survey was two-fold: (i) to evaluate the proposed criteria and accompanying guidance; and (ii) to freely comment on the overall approach, soundness, adequacy, consistency, and user-friendliness of the tool. Details about participants” affiliation, country of residence and years of experience in risk assessment were also collected (see Supplementary Table S2). The survey was created in the web-based software Survey and Report available *via* Karolinska Institutet. All participants gave informed consent to their personal information being collected and stored in accordance with the European General Data Protection Regulation (GDPR) and Swedish rules concerning the archiving of research data. Participation was voluntary and experts could withdraw at any time without giving any reason.

Detailed instructions for how to use the tool was provided to the participants via e-mail along with the three studies to be evaluated and a personal link to access the online survey. The participants were asked to first read the studies, and then evaluate their reliability and relevance with the SciRAP *in vitro* tool (online version 1.0). The list of the reliability criteria and relevance items of the tool version 1.0 used in the expert test round are provided as Supplementary Tables S3–S5). The reports generated as Excel files by the SciRAP tool (one per each study evaluated, i.e., three per participant) were collected *via* email. Participants were initially asked to complete the study evaluation and online survey within 3 weeks from receiving the test files. However, extensions were given in a few cases, as the timeframe for test completion was not considered critical to the purpose of our evaluation.

#### Selection of Round Test Participants

Experts within the field of *in vitro* toxicity testing and chemical risk assessment from regulatory authorities, academia, industry, and consultancy were invited to participate in the test round during the duration period stated above. The selection of participants was not randomized. Invitations were sent *via* email to individuals within our contact network in Europe, North America, South America, and Asia, also asking them to recommend additional experts who were contacted in turn. Participants were informed of procedures for handling personal data and gave their consent before agreeing to participate. In total, 31 participants with different affiliations and varying degree of experience completed the test round (anonymized information about the participant demographics is summarized in Supplementary Table S2).

#### Selection and Evaluation of the Test Studies

The participants were asked to evaluate the reliability and relevance of the same three *in vitro* studies using the SciRAP *in vitro* tool (online version 1.0):

• Study 1: Exposure of a human renal cell line to an aflatoxin and investigation of markers of senescence.
• Study 2: Exposure of HepG2 cells to a brominated flame retardant and investigation of induction of autophagy.
• Study 3: Exposure of human lung cell line to limonene oxidation products and investigation of inflammatory response.

The studies were selected based on the following considerations: (i) the study should evaluate some type of toxicity and be potentially relevant for hazard and risk assessment of human health effects; (ii) the study design should not be too complex, e.g., exposure only to a single substance and no mixture, using cell lines or primary cells, not several different assays used and reported in the same study; (iii) the study should be published open access and within the last few years; (iv) efforts were made to select three studies using different test systems (cellular models) and investigating different types of toxicity; (v) the intention was also to include studies with different levels of reporting, i.e., at least one well-reported study and one less well-reported study.

The purpose of the exercise was not to evaluate the quality/reliability of these individual studies, but to investigate how well the SciRAP *in vitro* tool performed for different types of studies and in handling different types of challenges in the study evaluation process. Our intent was to get a *first* indication of the practical use of the criteria, and a qualitative appraisal of the degree of agreement between expert when rating each criterion. While it is recognized that the selected studies do not capture the breadth of existing types of *in vitro* assays, we kept the number of studies to evaluate low to maintain a high level of participation among experts to get as many feedbacks as possible and pointers for further refinement of the tool.

### Data Analysis

#### Study Evaluation

Microsoft Excel was used to consolidate the results from each SciRAP report generated by the experts and to further analyze and compare the extent of expert agreement for each reliability criterion and relevance item per individual study and across studies. The variability in expert ratings for each reliability criterion and relevance item was evaluated semi-quantitatively, taking into account: (i) the number of rating categories observed for each reliability criterion (4 categories: “*fulfilled*,” “*partially fulfilled*,” “*not fulfilled*,” “*not determined*”) and for each relevance item (4 categories: “*directly relevant*,” “*indirectly relevant*,” “*not relevant*,” “*not determined*”); and (ii) the percentage of experts classifying a criterion or item in a given rating category. A decision tree (see Supplementary Figure S1) was developed to facilitate the identification and prioritization of criteria and items with high inter-expert variability; to this end, we applied simple decision rules for high variability based on cut-off values, whose exceedance would trigger further analysis and potential refinement. These cut-off values were not determined statistically, but were chosen arbitrarily given that our purpose was to develop a qualitative approach that would ensure a consistent and transparent handling of the information, in line with the aim and objectives of the work.

#### Online Survey

The Survey and Report tool (https://www.artologik.com/en/SurveyAndReport.aspx) exports survey results to an Excel file, which was then used for analysis of the survey data. In general, closed-ended questions were used in the survey, except in some cases where free comments were solicited from participants with open-ended questions. The subsequent qualitative analysis involved extracting and comparing information related to participant demographics and feedback on specific criteria and on the use of the tool (see previous section on *Expert Test Round Procedure*).

#### Prioritization Strategy

Both the expert evaluations and the online survey served as basis for refining the criteria and guidance items for *in vitro* studies on the SciRAP platform. We did not prioritize criteria or items where agreement between experts (i.e., when a criterion or item was allocated to the same rating category) was equal or greater than 80% but smaller than 100% (low variability decision rule). All criteria and items prioritized in at least one study according to the high variability decision rule (see decision tree) were evaluated for potential refinement (i.e., revise the criterion/item, or revise the accompanying guidance). However, based on the participant feedback from the survey, changes were also made to criteria that were not prioritized in the study evaluations.

## Results

The SciRAP approach for evaluating *in vitro* studies has been developed according to the same format and structure as the SciRAP approach for evaluating *in vivo* studies. The SciRAP *in vitro* tool is freely available via the SciRAP web-based platform (www.scirap.org).

### Expert Test Round (SciRAP Version 1.0)

#### Study Evaluation

**FIGURE 1:**
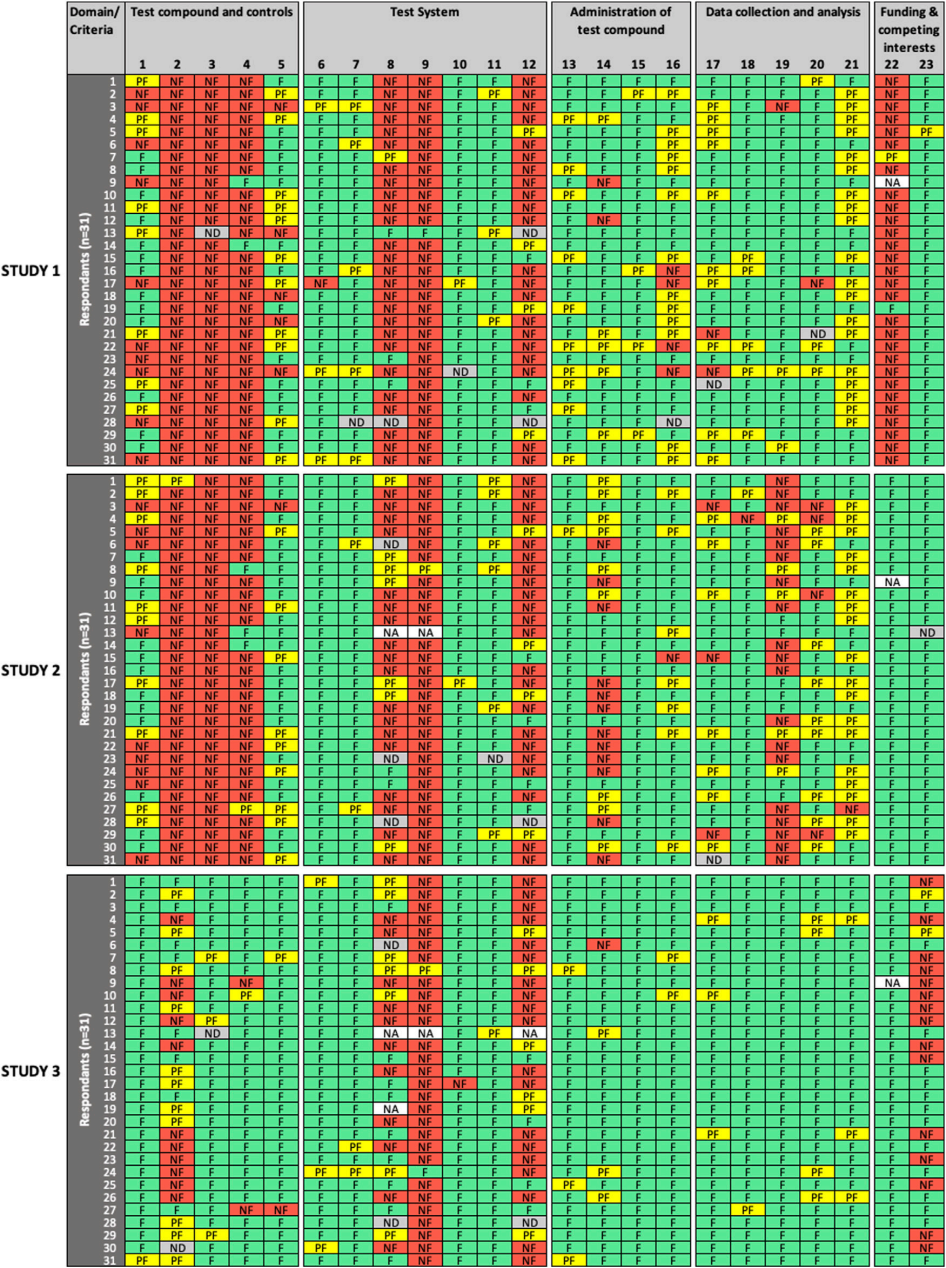
Results of the expert test round evaluations of reporting quality **(RQ)** of the three *in vitro* studies (*n* = 31 participants). Each row represents the evaluation by one participant; columns represent individual criteria. Green cells indicate criteria judged as “*fulfilled*” **(F)**, yellow cells indicate criteria judged as “*partially fulfilled*” **(PF)**, red cells indicate criteria judged as “*not fulfilled*” **(NF)**, grey cells indicate criteria left as “*not determined*” **(ND)**, and white cells indicate criteria removed by the participant as “*not applicable*” **(NA)**. *RQ* refers to how well the study design, methodology, conduct and results have been reported.

**FIGURE 2:**
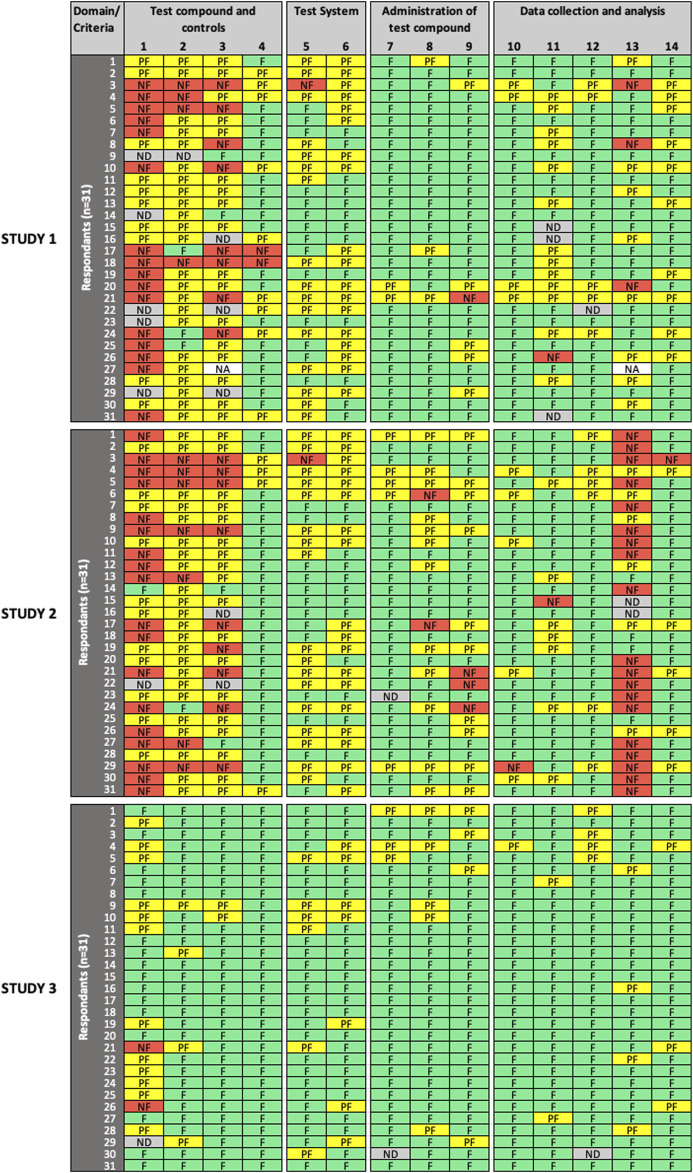
Results of the expert test round evaluations of methodological quality **(MQ)** of the three *in vitro* studies (*n* = 31 participants). Each row represents the evaluation by one participant; columns represent individual criteria. Green cells indicate criteria judged as “*fulfilled*” **(F)**, yellow cells indicate criteria judged as “*partially fulfilled*” **(PF)**, red cells indicate criteria judged as “*not fulfilled*” **(NF)**, grey cells indicate criteria left as “*not determined*” **(ND)**, and white cells indicate criteria removed by the participant as “*not applicable*” **(NA)**. *MQ* refers to the scientific soundness and appropriateness, including sensitivity, of the study design and methods used.

**FIGURE 3:**
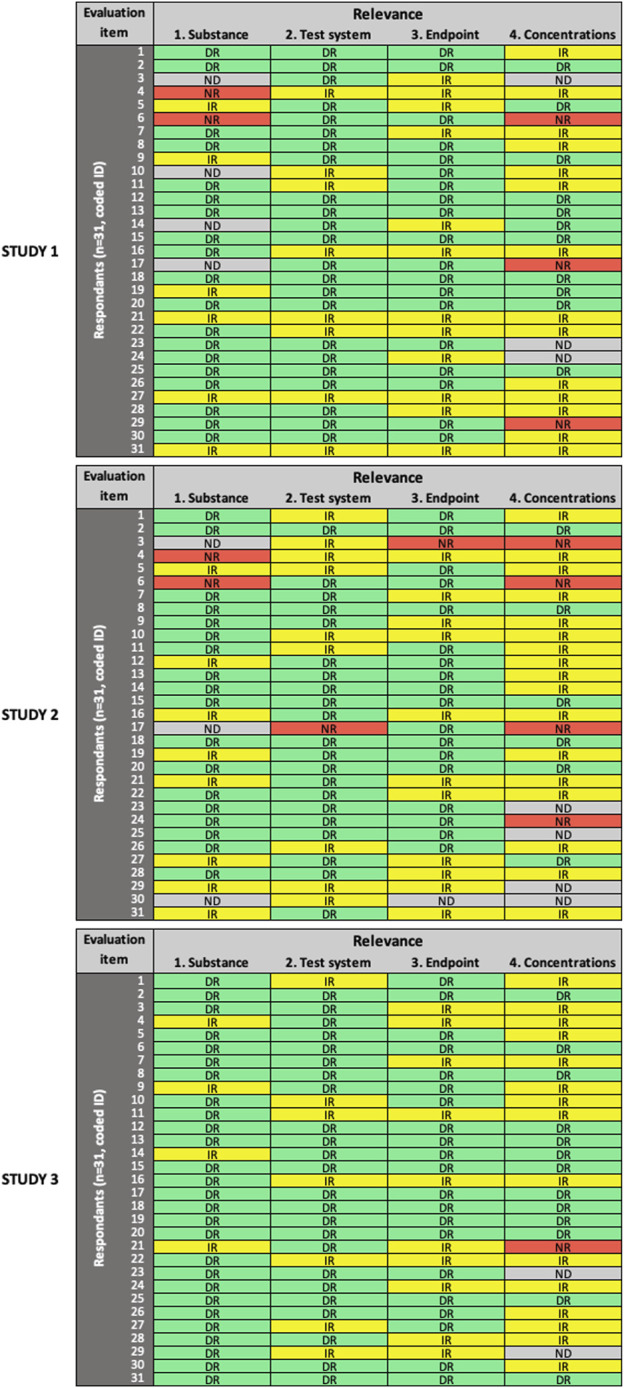
Results of the expert test round evaluations of the relevance of the three *in vitro* studies (*n* = 31 participants). Each row represents the evaluation by one participant; columns represent individual items. Green cells indicate items judged as “directly relevant” **(DR)**, yellow cells indicate items judged as “*indirectly relevant*” **(IR)**, red cells indicate items judged as “*not relevant*” **(NR)**, grey cells indicate items left as “*not determined*” **(ND)**.

All 31 participants completed the reliability and relevance evaluation of the three studies using the SciRAP *in vitro* tool (version 1.0) online. Figures 1–3 show the overview of expert ratings for each of the three studies evaluated in the expert round test for RQ and MQ reliability criteria, and relevance items, respectively (for detailed results see Supplementary Table S6). The results are presented according to the decision rules defined in the evaluation and prioritization strategy, i.e., based on cut-offs for low and high variability in expert ratings. Cases where no variability was observed are referred to as “consensus.”

For RQ evaluation (Figure 1), low variability in expert ratings was observed for a number of evaluated criteria in all domains for one study (criteria #2, 5, 8, 15, 16, 17, 19, 20, 21), for two studies (criteria #3, 6, 11, 13, 20, 23), or for all three studies (criteria #4, 7, 9, 10, 15, 18, 22) evaluated. In addition, consensus was observed for criteria #2, 3, 6, 19 in one study, and criteria #15, 22 in two studies. High variability in expert ratings was observed in the evaluation of criteria #2 (“test compounds and controls” domain), #14 (“administration of test compound” domain), and #23 (“funding and competing interests” domain) in one study; criteria #1, 5 (“test compounds and controls”), #8 (“test system” domain), #16 (“administration of test compound”), #17 (“data collection and analysis” domain) in two studies; and for criterion #12 (“test system”) in all three studies.

For MQ evaluation (Figure 2), low variability in expert ratings was observed for criterion #7 (“administration of test compound”) and criteria #10, 12 (“data collection and analysis”) in all three studies evaluated; and for criteria # 4 (“test compounds and controls”), #8, 9 (“administration of test compound”), and #14 (“data collection and analysis”) in two studies. For Study 3, low variability in expert ratings was also observed for all criteria, except for criterion #1 (“test compounds and controls”), which had a high variability in expert ratings in all evaluated studies. Consensus was only observed for criterion #4 in Study 3. High variability in expert ratings was observed in Study 1 and Study 2 for criteria #2, 3 (“test compounds and controls”), #5, 6 (“test system”), #11, 13 (“data collection and analysis”), as well as for criteria #8, 9 (“administration of test compound”) but for Study 2 only.

SciRAP includes a function that allows the evaluator to remove a criterion if considered not adequate or irrelevant in the context of the evaluation (e.g., due to the study type or substance. A removed criterion is then displayed as “*not applicable*” (NA) in the colour profile by the system. In removing a criterion, the expert avoids judging it, a situation that should not be confused with rating a criterion as “*not determined*,” which would typically occur in case of poor reporting, when the evaluator considers that there is not sufficient information at hand to make a decision, i.e., to determine if the criterion is “*fulfilled*,” “*partially fulfilled*,” or “*not fulfilled*”. Four participants (13%) chose to remove RQ criteria (9 instances) and MQ criteria (2 instances), and the removed criteria were not taken into account in the analysis. Justification was only provided in two cases, in particular for MQ criterion #13, where the expert justified removal because cytotoxicity was an endpoint evaluated in Study 1. Interestingly, significant disagreement was observed among raters for that criterion, which could have been misinterpreted in that specific case.

For the evaluation of relevance (Figure 3), low variability in expert ratings was observed only for item #1 (“substance” domain) in Study 3. In general, more variability was observed in all domains across the three studies, compared to RQ and MQ evaluation. High variability in expert ratings was observed for item #4 (“concentrations” domain) in all three studies; item #1 (“substance”) in Study 1 and Study 2; and item #2 (“test system” domain) and item #3 (“endpoint” domain) in Study 2.

#### Online Survey

Thirty experts (97%) participated to the online survey. Participants were in general positive with the tool. They felt that the SciRAP criteria were “*appropriate*” (n = 26, 87%) or “*somewhat appropriate*” (*n* = 4, 13%) for evaluating RQ of *in vitro* studies. For MQ evaluation, 77% (*n* = 23) of respondents considered that criteria were “*appropriate*,” and 23% (*n* = 7) that they were “*somewhat appropriate*.” However, only 67% (*n* = 20) of participants considered that the relevance items were “*appropriate*.” Several participants highlighted that evaluation of relevance was challenging without the assessment/application context, in particular when judging of the relevance of the concentrations used or the susbtances tested. In the SciRAP 1.0 version, guidance was only available for MQ, a feature that a majority of participants found “*useful*” (n = 23, 77%). Participants also largely found that the color-coding feature of the tool was “*useful*” (n = 28, 93%).

Roughly one third of the participants took the opportunity to freely comment on different aspects of the reliability criteria and relevance items, and on the tool in general. Some common suggestions were:

• Provide guidance to support the evaluation of RQ criteria, in particular in the “*test compounds and controls*,” “*test system*,” and “*data collection and analysis*” domains.
• Improve existing guidance for MQ criteria, in particular in the “*test compounds and controls*” and “*test system*” domains.
• Add specific evaluation criteria for RQ and/or MQ, e.g., to address reproducibility issues and blinding issues; the use of positive controls; or reporting bias.
• Improve existing guidance for relevance items.

### SciRAP *in vitro* Tool (Version 2.0)

The expert feedback (which includes the evaluation results and online survey) was primarily considered qualitatively to help us improve the tool as we see fit in terms of general desired characteristics such as adequacy, user-friendliness, and flexibility. All RQ and MQ criteria prioritized during study evaluation (i.e., according to the decision tree) were either revised or had their guidance items refined (for MQ only). Some criteria (RQ#4, 6) that were not prioritized in the study evaluation were also revised, based on the expert feedback in the online survey. Revisions consisted effectively in slight reformulation of the criteria. The details of the prioritization strategy are presented in Supplementary Table S7.

#### SciRAP Reliability Criteria (Version 2.0)

The SciRAP *in vitro* tool (version 2.0) consists of 24 criteria for “*reporting quality*” and 16 criteria for “*methodological quality*” (Tables 1, 2) based on the expert test round. Supplementary Tables S8–S9 present the details of the refinements that were made to develop version 2.0 from version 1.0, i.e., which criteria were prioritized, what action was triggered with accompanying justification. New guidance for RQ criteria and revised guidance for MQ criteria version 2.0 will be soon available online.

**TABLE 1:** SciRAP criteria for assessing reporting quality of *in vitro* toxicity studies (version 2.0).

SciRAP reporting quality criteria per evaluation domain (version 2.0)	
***Test compound and controls***	
1.	The chemical name or other identification, such as CAS-number, of the test compound was given
2.	The purity of the test compound was stated or is traceable according to information given regarding manufacturer and lot/batch number. In case of mixtures, the composition of different constituents was stated
3.	The solubility of the test compound was described
4.	The solvent (vehicle) was described
5.	It was stated that a solvent (vehicle) control was included
***Test System***	
6.	The test system (e.g., cell line/cells/tissue/organ/embryo/sub-cellular fractions) was described
7.	The source of the test system was stated
8.	The metabolic competence, i.e., competence of the test system to metabolize the test compound into an active metabolite was described
9.	The number of cell passages of the cell line used, was stated. (Remove this criterion if the study was not conducted in a cell line.)
10.	Composition of media was described, including use of serum, antibioticsetc.
11.	Incubation temperature, humidity, and CO2 concentration were described
12.	Measures taken for avoiding or screening for contamination by *mycoplasma*, bacteria, fungi and virus were described
***Administration of test compound***	
13.	The administered dose levels or concentrations were stated
14.	Cell density or number of cells used during treatment was described. (Remove this criterion if the study was not conducted in a cell line.)
15.	The duration of treatment was stated
16.	The number of replicates per dose level/concentration or the number of times the experiment was repeated was stated
***Data collection and analysis***	
17.	The tests and/or analytical methods used were sufficiently described to allow for evaluation of reliability of results
18.	The time points for data collection were stated
19.	It was stated that the effect of the test compound on cytotoxicity was measured
20.	All results were clearly presented
21.	The statistical methods and software used were described
***Funding and competing interests***	
22.	The funding sources for the study were stated
23.	Any competing interests were disclosed or it was explicitly stated that the authors did not have any competing interests
***Other***	
24.	Was all information that is indispensable for evaluating the reliability of data given? This includes information on the test compound and controls, test system, study design or study performance

**TABLE 2:** SciRAP criteria for assessing methodological quality of *in vitro* toxicity studies (version 2.0).

SciRAP methodological quality criteria per evaluation domain (version 2.0)	
***Test compound and controls***
1.	The chemical name or other identification, such as CAS-number, of the test compound was given
2.	The purity of the test compound was stated or is traceable according to information given regarding manufacturer and lot/batch number. In case of mixtures, the composition of different constituents was stated
3.	An appropriate solvent (vehicle) was used that is not expected to interfere with the results of the study at the concentration used
4.	A solvent (vehicle) control was included
5.	An appropriate positive control was included, and the expected result was observed from this treatment
***Test System***
6.	A reliable and sensitive test system (e.g., cell line/cells/tissue/organ/embryo/sub-cellular fractions) with metabolic competence, if relevant, was used for investigating the test compound and endpoints
7.	Conditions for cultivation and/or maintenance of the cell line/cells/tissue/organ/embryo/sub-cellular fractions (incubation temperature, humidity, CO2 concentration, media used, number of cell passages, control of contamination) were appropriate
***Administration of the test compound***
8.	The duration of exposure was suitable for the test system and investigated endpoints
9.	The concentrations used were suitable for the test system and investigated endpoints
10.	The test conditions during and after exposure to the test compound were suitable (media and serum used, cell density, incubation temperature, humidity, CO2 concentration)
***Data collection and analysis***
11.	Reliable and sensitive tests and/or analytical methods were used for investigating the endpoints
12.	Sufficient numbers of replicates or repetitions of the experiment were used to generate reliable and valid results
13.	Measurements were collected at suitable time points in order to generate sensitive, valid and reliable data
14.	Cytotoxicity was measured and the test compound did not cause cytotoxicity that significantly affected the results
15.	The statistical methods were clearly described and do not seem inappropriate, unusual or unfamiliar
***Other***	
16.	Are there any other aspects of study design, performance or reporting that influence reliability?

Five (22%) RQ criteria (#1, 4, 5, 6, 8) and 4 (27%) MQ criteria (#3, 4, 5, 6) were revised based on the expert test round. Criteria were also added based on expert feedback in the online survey: a new MQ criterion (use of an appropriate positive control), and a new RQ open comment (“Was all information that is indispensable for evaluating the reliability of data given”). In general, the outcome of the expert test round showed a clear need to add guidance for several RQ criteria (n = 9, 39%), as well as to refine existing guidance for a large number of MQ criteria (n = 11, 73%), without necessarily calling for the need to revise the formulation of the criteria. However, some criteria required both reformulation and revision of the guidance (RQ criteria #1, 5, 8; MQ criteria #3, 4, 5, 6); in particular criteria that include several different aspects (MQ criteria #5, 6) tend to open up for higher variability between raters, since the room for interpretation widens. However, we have tried to find an acceptable balance between having only single-aspect criteria and not having too many criteria. Raters are also encouraged to use the “comment” function for each criterion to flagship any particular aspect deemed relevant. For some criteria we decided to improve the online guidance by cross-checking with the corresponding information in the OECD Test Guidelines (OECD, 2017) and/or the GIVIMP (OECD, 2018) to be better in line with the OECD terminology and requirements, e.g., RQ criterion #4 and MQ criterion #4 (control/vehicle); MQ criteria #8, 9 (test concentrations and conditions); and RQ criterion #12 (contamination sources) and MQ criterion #11 (statistical power calculations).

#### SciRAP Relevance Items (Version 2.0)

The SciRAP *in vitro* tool (version 2.0) consists of 4 items for evaluating relevance (Table 3). We decided not to revise the relevance items or accompanying guidance at this stage of development of the tool. Therefore, the relevance items (version 1.0) remain unchanged. Consistent with the feedback from the expert round test, judging whether the concentrations used or the endpoints investigated as “*directly relevant*,” “*indirectly relevant*,” or “*not relevant*” is difficult in absence of specific information about the hazard or risk assessment context. The purpose of the SciRAP items for evaluating the relevance of individual *in vitro* studies is to provide a structure for considering how the study contributes information that is relevant for the question to be answered or the problem at hand. It is important to note that all listed relevance items do not have to be judged as relevant for the study to serve as evidence or supportive evidence in risk assessment.

**TABLE 3:** SciRAP items for assessing relevance of *in vitro* toxicity studies for health hazard or risk assessment (tool version 2.0).

SciRAP relevance items per evaluation domain (v2.0)	
***Test compound***
1. The identity of the tested substance
***Test System***
2. The test system used
***Endpoint***
3. The endpoint studied
***Concentrations***
4. The concentrations used

### Export and Interpretation of Evaluation Results Using the SciRAP Tool

**FIGURE 4:**
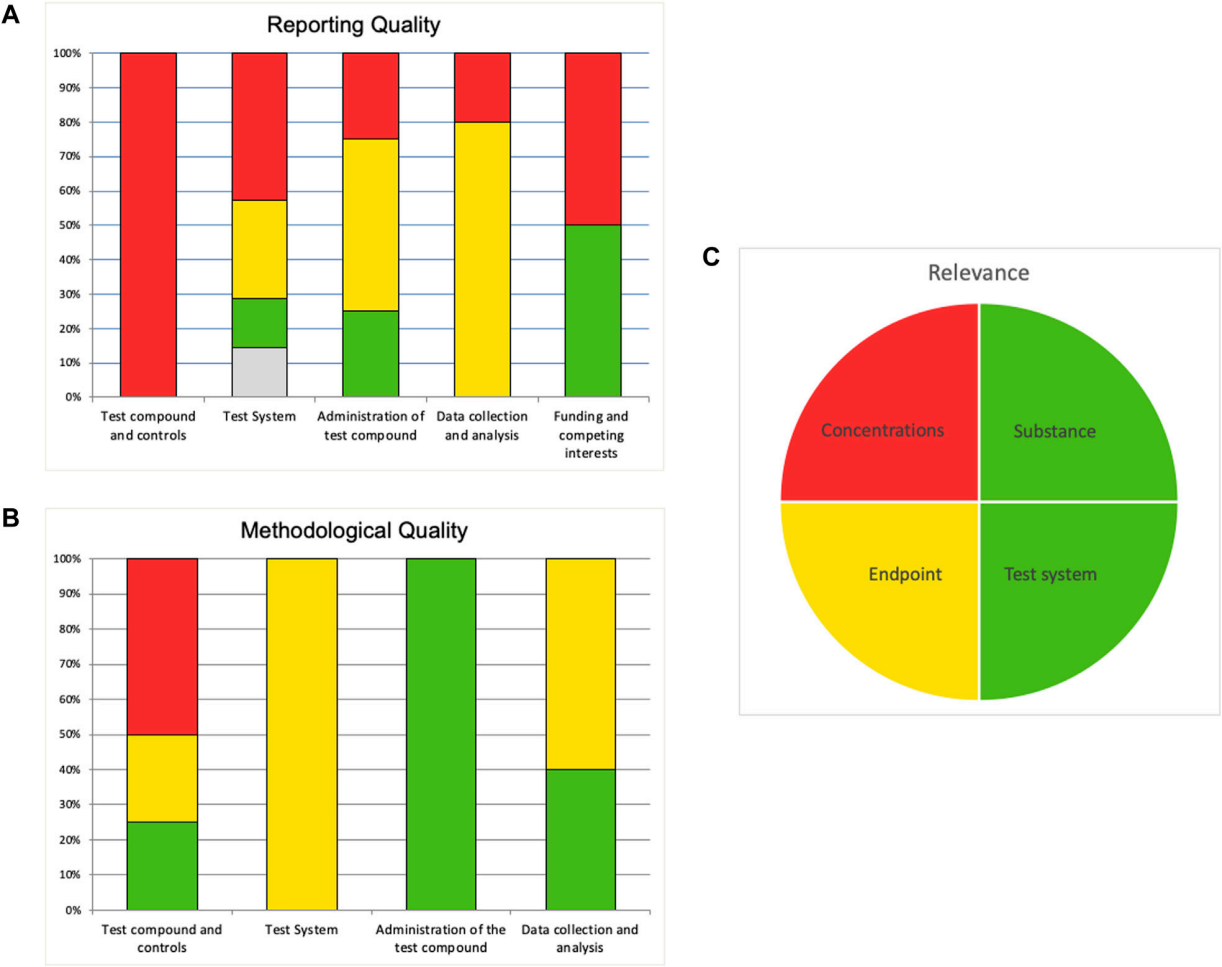
Example of a colour profile for an *in vitro* study evaluated in the SciRAP *in vitro* tool. “*Reporting quality*” **(A)** and “*Methodological quality*” **(B)** are illustrated in bar diagrams. Each bar shows the percentage of criteria judged as “*fulfilled*” **(green)**, “*partially fulfilled*” **(yellow)**, “*not fulfilled*” **(red)** and “*not determined*” **(grey)**, taking into account if the weight has been increased for any criteria. Criteria that have been removed are not included. Evaluation of relevance is illustrated in a pie chart **(C)**. If an item was evaluated as “*directly relevant*” it is shown as green, if it was judged as “*indirectly relevant*” it is shown as yellow, and if it was judged as “*not relevant*” it is shown as red.

When the evaluation is completed in the SciRAP tool online, a summary of the evaluation may be exported to an Excel file. Similar to the SciRAP tool for evaluating *in vivo* studies (Beronius et al., 2018), the Excel file contains a summary and a colour profile giving an overview of the RQ and MQ (reliability) of the study, presented as bar charts, as well as relevance, presented as a pie chart (Figure 4A–C). In the colour profile, RQ and MQ criteria are grouped into different categories represented by separate bars for the “*test compound and controls*,” “*test system*,” “*administration of the test compounds*,” and “*data collection and analysis*” domains; and for RQ also “*funding and competing interests*” domain. Each bar shows the percent of criteria in that category judged as “*fulfilled*” (green), “*partially fulfilled*” (yellow), “*not fulfilled*” (red) and “*not determined*” (grey). Similarly, the four items for considering relevance are shown in the pie chart as green if it was judged as “*directly relevant*,” yellow if it was judged as “*indirectly relevant*,” and red if it was judged as “*not relevant*”.

As described briefly above, the SciRAP tool includes a function to increase the weight of a criterion (from the default value of 1–1.5) if the assessor deems it to be specifically critical to the evaluation at hand. This can for example be based on the type of toxicity or substance investigated. Individual criteria that are considered irrelevant for the evaluation can be removed from the evaluation. these functions introduce flexibility to the SciRAP tool, it is important to note that increasing the weight of criteria or removing them will impact the SciRAP evaluation readout. The relevance items cannot be weighed up or removed. If a criterion has been given higher weight this will affect its representation in the colour profile as it will be represented by a larger area of the chart. Criteria that have been removed are also removed from the representation in the charts, i.e., the colour profile will be based only on the number of criteria considered in the evaluation. For a more thorough description of these functions of the SciRAP tool and their effect on the evaluation read-out, see Beronius et al. (2018).

In addition to the qualitative presentation of the evaluation results in the colour profile, the SciRAP tool also calculates a numerical score for RQ and MQ, respectively. Eq. 1 shows the calculation of the SciRAP score where F  is the number of “*fulfilled*” criteria, PF  is the number of “*partially fulfilled*” criteria and T  is the total number of criteria, excluding criteria that have been removed. The calculation of the score takes into account the weight attributed to individual criteria, i.e., each criterion is multiplied by its weight.

#### Equation 1

$$\mathrm{SciRAP}\;\mathrm{score}=\frac{F+\left(\mathrm{PF}\text{*}0.5\right)}{T}*100$$ (1)

The SciRAP score can have a value ranging from 0 (all criteria are judged as “*not fulfilled*”) to 100 (all criteria are judged as “*fulfilled*”). The numerical score should be interpreted with caution and always in combination with considering the colour profile. The score only gives an indication of the % fulfilled criteria and will not inform on the particular strengths or limitations of a study, which may have different impacts on the overall reliability depending on their nature. Importantly, criteria judged as “*not fulfilled*” and as “*not determined*” will have the same impact on the score.

## Discussion

An overarching aim of the SciRAP initiative is to facilitate structured use of relevant toxicity data for regulatory hazard and risk assessment of chemicals. Here we present the work to develop the SciRAP tool for evaluation of *in vitro* data, version 2.0, including consideration of feedback from intended end users within agencies, industry and academia. Tools for evaluation of *in vitro* data for use in hazard and risk assessment are becoming increasingly important as focus on reducing animal testing and developing pathway-based Integrated Approaches to Testing and Assessment (IATA) for regulatory purposes increases. The intention is that the SciRAP approach to data evaluation should be flexible and applicable for use in different assessment contexts and across different regulatory frameworks. SciRAP provides criteria for the evaluation of reliability and relevance of *in vitro* studies for regulators and risk assessors as well as researchers. The criteria presented here can also be used by reviewers and editors to inform the review process for scientific publication to increase the reliability and reproducibility of studies.

The SciRAP *in vitro* tool version 1.0 was developed based on requirements and recommendations in relevant OECD Test Guidelines in order to ensure coverage of aspects of study design, conduct and reporting that have international acceptance and agreement. Most of the expert test round participants found the tool useful and adequate for rating RQ and MQ of *in vitro* toxicity studies, which can be tied to the tools strengths in terms of flexibility, adequacy, and user-friendliness. The participants, who represented intended end-users from regulatory authorities, industry, and academia, furthermore provided valuable feedback on the content and application of the tool that was useful for refinement and development of version 2.0. It was difficult to assess the four items used for relevance evaluation in this test round, as it would require context in the form of a specific problem formulation. However, the participants could still provide comments on the general format and content of the relevance items, which will be taken into account in future refinements of the tool.

One limitation of the expert test round is that it is not entirely representative of the heterogeneity in *in vitro* studies one would usually see at desk level in real-life hazard/risk assessment practice (e.g., short/simple vs. long/complex study designs; academic peer-review studies vs. industry studies; specificity of the application/regulatory context; etc). Indeed, the three selected *in vitro* studies were intentionally chosen to have relatively “simple” study designs to not deter experts from participating. Thus, it is acknowledged that these studies are not representative of the wide range of different *in vitro* study designs available and commonly applied. The issue of generalizability of the SciRAP tool (or any evidence appraisal tool) is of importance. There is a need to strike a balance in terms of providing criteria with enough specificity to promote consistency between evaluators, but generic enough to allow for flexibility so that the criteria can be applied to different data typologies, test compounds, study designs, and endpoints. This is a recognized challenge, based on our experience in formulating evaluation criteria for *in vivo* data (Beronius et al., 2018) that should be applicable to very variable study designs, using a myriad of different models and analytical methods, and investigating very heterogenous sets of toxicity endpoints. These aspects can be addressed in future developments of the SciRAP tool, as future uses in different settings and application contexts will provide further insights that can be used to refine the tool.

It should also be noted that study evaluation can be conducted on different levels. In some cases, it is possible to evaluate the study as a whole, for example if the specific endpoint of interest is the only parameter investigated in the paper and there is only one model or method used. However, most commonly several endpoints are investigated in the same study, sometimes using different models and methods. In such cases, it is necessary to evaluate the specific experimental design, conduct and reporting of the methods used to investigate the endpoint of interest. As a result, a single study may need to be evaluated for RQ, MQ, and relevance several times for different endpoints of interest, the level of granularity of the evidence appraisal depending on the specificity of the risk assessment context and aim.

One participant pointed out that there are some redundancies between RQ and MQ criteria. However, this is an intentional feature of the tool, with the idea that end-users should be able to evaluate RQ and MQ separately.

The non-randomized selection of study participants may also be considered a limitation, as it resulted in an over-representation of European participants compared to non-European participants. In addition, participants from Switzerland were over-represented compared to participants from the rest of Europe. Ideally, a more balanced geographical distribution of participants would be desirable to ensure a better generalizability of the results, and this will be taken into account in further developments of the SciRAP *in vitro* tool. However, while cultural and institutional differences in hazard/risk assessment practice across regions may contribute to shape risk assessors experience and work at desk level, we assumed that the geographical representation in the expert test round is not a critical shortcoming at this stage of development of the SciRAP *in vitro* tool, since data quality requirements (as laid down by internationally accepted guidelines and standards such as the OECD Test Guidelines, regional or national standards such as CEN or DIN) and risk assessor’s needs should not be significantly different across various geographical areas. Importantly, we were able to include representatives from authorities, industry and academia to include perspectives that may differ between these sectors, as well as evaluators with different levels of experience (see Supplementary Table S2).

Another particular challenge for several participants was to evaluate the statistical methods applied (RQ criterion #21). We acknowledge that this criterion is often one that is difficult to address and requires expertise in statistical principles and methods. At the same time, it is an aspect that is important to consider during study evaluation as it has bearing on the reliability of study results.

Some participants raised the question whether SciRAP should not include analysis of risk of bias, a methodology used to assess internal validity of studies in systematic reviews and meta-analyses (e.g., NHRMC, 2019; Higgins et al., 2021). Assessment of risk of bias and of quality/reliability are related but distinct concepts (NTP, 2019). SciRAP does not integrate risk of bias considerations, therefore there are no criteria that specifically target risk of bias. Also, formulating one-size-fits-all criteria that capture both risk of bias and reliability considerations would blur the line between the two approaches, which is not desirable. Risk of bias and reliability evaluations may not target the same “quality” dimension of the object under consideration, or only partially if the intrinsic quality/internal validity domains overlap, and may involve different decision rules when rating a criterion. This may represent a source of ambiguity, which may in turn increase inter-rater variability. An example of this relates to the high variability observed in expert ratings for RQ criterion #23 (conflict of interest (COI) statement) in study 3. If the evaluator approaches the criterion with a “risk of bias mindset,” declaration of a COI may be intrepreted negatively since this is a bias that can bear on the validity of the study under evaluation, leading to rating the criterion as “*not fulfilled*,” whereas explicit statement of absence of COI would be rated as “*fulfilled*.” If the evaluator focuses on the quality of the reporting (what is being asked here), declaration of a COI (or declaration of not having a COI) would be rated (equally in fact) as “*fulfilled*”, whereas the absence of reporting a COI would be rated as “*not fulfilled*.” In this case we did not revise the criterion, but proposed that guidance be refined to better explain what is expected from the evaluator, in order to reduce potential for misinterpretation and inter-expert variability.

The SciRAP approach was initially developed with the purpose of promoting and improving structured and transparent evaluation of evidence in regulatory hazard and risk assessment in the EU. It is therefore based around evaluation of reliability and relevance since this is the focus in EU chemicals regulation. However, as systematic review methodology is being increasingly incorporated in chemical risk assessment practice and weight-of-evidence analyses/frameworks in the regulatory context (e.g., EFSA, 2015; Whaley et al., 2016; Hoffmann et al., 2017; Schaefer and Myers, 2017; Radke et al., 2020; Roth et al., 2020) it is relevant to discuss the potential alignment of SciRAP with systematic review methodology. We have previously compared the SciRAP *in vivo* tool to available approaches for evaluating risk of bias (Waspe et al., 2021). The investigation showed that the output from the SciRAP *in vivo* tool evaluation could be readily translated into conclusions for risk of bias domains analysis. Formal approaches for evaluating risk of bias of *in vitro* toxicity studies are very limited (NTP, 2016, 2019). Further research is needed to explore the crosstalk between existing approaches to data quality/reliability and relevance and internal validity/risk of bias analysis.

The SciRAP *in vitro* tool, version 2.0. is freely available online at www.scirap.org and provides a means to ensure structured and transparent evaluation of *in vitro* data for hazard and risk assessment. Although the scientific soundness and applicability of the criteria and tool was evaluated in the expert test round described here, it is envisioned that they will undergo further refinement based on future use and we welcome feedback from users. This may include, for example:

• Adjustments to specific uses or applications in a regulatory hazard or risk assessment context, such as in the case of nanomaterials, for which specific criteria for evaluation of phys-chem parameters, exposure, and controls have been proposed (Fernández-Cruz et al., 2018). The SciRAP platform already contains a specific tool version to be used for ecotoxicity studies on nanomaterials; work is ongoing to further extend SciRAP to *in vitro* and *in vivo* studies on nanomaterials.
• Adjustments to align with development of automated text mining tools. The feasibility of using automated data mining to facilitate the assessment of RQ in SciRAP *in vitro* tool is currently under evaluation.
• Improvement and revision of guidance for RQ and MQ.

## Data Availability

The original contributions presented in the study are included in the article/Supplementary Material, further inquiries can be directed to the corresponding author.
