# Supplementary material for: Development of the SciRAP Approach for Evaluating the Reliability and Relevance of in vitro Toxicity Data
Source: Front Toxicol. 2021 Oct 15;3:746430. doi: 10.3389/ftox.2021.746430 (PMC8915875; doi:10.3389/ftox.2021.746430)
Supplement: Supplementary file 1 [file Table1.docx]

Supplementary Material

**Supplementary Table S1.** Online survey, structured questionnaire.

| Personal information |
| --- |
| 1. I hereby give my consent that my personal data (name, e-mail, affiliation and years active in the field of research and/or risk assessment) is collected for the purpose of this study. I confirm that I have read and understand the information about handling of personal data in this study provided in the invitation to join the study. I understand that my participation is voluntary and that I am free to withdraw at any time without giving any reason. |
| 1. Your name |
| 1. What is your country of residence? |
| 1. What is your affiliation?  - Academia - Government/Authority - Industry - Consultancy - Other, please specify |
| 1. Name of employer: |
| 1. How many years’ experience do you have in the area of conducting *in vitro* research?  - None - <1 - 1-5 - 5-10 - 10-15 - >15 |
| 1. How many years’ experience do you have in the area of risk assessment of chemicals?  - None - <1 - 1-5 - 5-10 - 10-15 - >15 |
| Study evaluation |
| 1. Based on your evaluation according to SciRAP, how would you categorise the reliability of Study 1/2/3? (*This question is repeated 3 times, once for each study*).  - Reliable – The study is sufficiently well reported to allow for evaluation, i.e. critical information about study design, conduct and results is included. Most SciRAP criteria for reporting quality were evaluated as “fulfilled” or “partially fulfilled”. All SciRAP criteria for methodological quality were evaluated as “fulfilled” or “partially fulfilled”. Single criteria may have been evaluated as “not fulfilled” or “not determined” if not judged to significantly influence the reliability of results or to bias results against the null. - Reliable with restrictions – The study is sufficiently well reported to allow for evaluation, i.e. critical information about study design, conduct and results is included. Most SciRAP criteria for reporting quality were evaluated as “fulfilled” or “partially fulfilled”. The study is generally well designed and performed but some minor flaws in the methodology may be present that could have influenced the results or biased the results against the null. - Not reliable – The study is sufficiently well reported to allow for evaluation, i.e. critical information about study design, conduct and results is included. Most SciRAP criteria for reporting quality were evaluated as “fulfilled” or “partially fulfilled”. Critical SciRAP criteria for methodological quality were evaluated as “not fulfilled”. The study has clear flaws, primarily in how it was designed and performed, which are likely to significantly influence the reliability of results or to bias results against the null. OR the study has serious flaws in reporting and it is not likely that gaining access to missing information will improve the reliability of the study. - Not assignable – The study is too poorly reported; critical information about the study design or conduct that is needed to make an assessment of the study is missing. |
| 1. Based on your evaluation according to SciRAP, how would you categorise the relevance of Study 1/2/3? (*This question is repeated 3 times, once for each study*).    - Directly relevant - the test system, concentrations tested and endpoints measured are relevant for measurement of human health outcomes or modes of action/key events related to human health outcomes.    - Indirectly relevant - the test system, concentrations tested and endpoints measured are partly relevant for measurement of human health outcomes or modes of action/key events related to human health outcomes. However, there are e.g. species or cell type differences reducing the relevance of the model.    - Not relevant – the test system, concentrations tested and/or endpoints measured are not relevant for measurement of human health outcomes or modes of action/key events related to human health outcomes. |
| The SciRAP approach |
| 1. How much time do you estimate you spent on average on evaluating each study using the SciRAP tool?    - <1 hour    - 1-2 hours    - >2 hours |
| 1. Is this amount of time reasonable?  - Yes - No - Comment: |
| 1. In your opinion, are the criteria for evaluating reporting quality appropriate for the evaluation of in vitro studies?  - Yes - Somewhat - No - Comment: |
| 1. Please specify any additional criteria that could be important for evaluation of reporting quality of in vitro studies for health risk assessment. |
| 1. In your opinion, are the criteria for evaluating methodological quality appropriate for the evaluation of in vitro studies?  - Yes - Somewhat - No - Comment: |
| 1. Please specify any additional criteria that could be important for evaluation of methodological quality of in vitro studies for health risk assessment. |
| 1. In your opinion, are the criteria for evaluating relevance appropriate for the evaluation of in vitro studies?  - Yes - Somewhat - No - Comment: |
| 1. Please specify any additional criteria that could be important for evaluation of relevance of in vitro studies for health risk assessment. |
| 1. Did you find the guidance provided with the criteria helpful?  - Yes - Somewhat - No - Comment: |
| 1. Did you find the web-based colour-coding tool useful in the application of the SciRAP evaluation criteria for reporting quality, methodological quality and relevance?  - Yes - Somewhat - No - Comment: |
| 1. Are you currently using any specific method for evaluating the quality of data for risk assessment?  - Yes, please specify which one(s) - No |
| 1. Compared to the approach for data evaluation you are currently using (even if no specific method is used), how does SciRAP compare concerning  - Facilitating transparency in the evaluation? - Dependence on expert judgment and need for expertise? - Accuracy of the evaluation? - Consistency between evaluations? - User-friendliness? - Time required?   Answer options: *Better than current approach*; *the same as current approach*; *Not as good as current approach*; *Don’t know* |
| 1. Please provide any other comments that could help further develop and improve the study evaluation criteria, web-based tool and/or the SciRAP website. |
