# Supplementary material for: Development of the SciRAP Approach for Evaluating the Reliability and Relevance of in vitro Toxicity Data
Source: Front Toxicol. 2021 Oct 15;3:746430. doi: 10.3389/ftox.2021.746430 (PMC8915875; doi:10.3389/ftox.2021.746430)
Supplement: Supplementary file 2 [file Table8.docx]

Supplementary Material

**Supplementary Table S8**. Changes made to SciRAP *in vitro* reporting quality criteria (version 1.0) based on the expert test round. Criteria marked in blue had high inter-expert variability for one of the 3 studies evaluated by the experts in the test round, criteria marked in green had high variability for 2 or all 3 studies evaluated in the test round. There was no guidance to support evaluation of the reporting quality in version 1.0, therefore new guidance will be added for all reporting quality criteria in version 2.0.

| **Version 1.0 – February 2018** | **Adjustments for Version 2.0 – 2021**  *(If nothing stated here no change to v1.0 criterion is made)* |
| --- | --- |
| **Test compound and controls** | |
| 1. The chemical name, ID or CAS-number of the test compound was given. | The chemical name or other identification, such as CAS-number, of the test compound was given.  *This criterion was adjusted based on the high variability in expert ratings observed in the study evaluation.* |
| 1. The purity of the test compound was stated or is traceable according to information given regarding manufacturer and lot/batch number. In case of mixtures, the composition of different constituents was stated. | *No change; high inter-expert variability should be reduced by added guidance.* |
| 1. The solubility of the test compound was described. |  |
| 1. The vehicle was described. | The solvent (vehicle) was described.  *Though not prioritized in the study evaluation, this criterion was adjusted based on expert feedback in the online survey and cross-checking against terminology in relevant OECD test guidelines.* |
| 1. It was stated that an untreated or vehicle control was included. | It was stated that a solvent (vehicle) control was included.  *This criterion was adjusted based on the high variability in expert ratings observed in the study evaluation and also based on the expert feedback in the online survey.* |
| **Test System** | |
| 1. The test system (cell line / cells/ tissue / organ / embryo) was described. | The test system (e.g., cell line / cells/ tissue / organ / embryo / sub-cellular fractions) was described.  *Though not prioritized in the study evaluation, this criterion adjusted based on expert feedback in the online survey.* |
| 1. The source of the test system was stated. |  |
| 1. Metabolic competence of the test system was described. | The metabolic competence, i.e., competence of the test system to metabolize the test compound into an active metabolite was described.  *This criterion was adjusted based on the high variability in expert ratings observed in the study evaluation and also based on the expert feedback in the online survey.* |
| 1. The number of cell passages of the cell line used was stated. (Remove this criterion if the study was not conducted in a cell line.) |  |
| 1. Composition of media was described, including use of serum, antibiotics, etc. |  |
| 1. Incubation temperature, humidity, and CO2 concentration were described. |  |
| 1. Measures taken for avoiding or screening for contamination by mycoplasma, bacteria, fungi and virus were described. | *No change; high inter-expert variability should be reduced by added guidance. Guidance item should mention that aseptic techniques are used or that tests for contamination by i.e., mycoplasma are done. Also reference to GIVIMP section 4.4 and 5.7 can be added.* |
| **Administration of test compound** | |
| 1. The administered dose levels or concentrations were stated. | The future guidance item will contain text specifically addressing considerations of measured concentrations, as well as administered dose.  *Adjustments to the guidance based on feedback during the review process.* |
| 1. Cell density or number of cells used during treatment was described. (Remove this criterion if the study was not conducted in a cell line.) | *No change; high inter-expert variability should be reduced by added guidance.* |
| 1. The duration of treatment was stated. |  |
| 1. The number of replicates per dose level/concentration or the number of times the experiment was repeated was stated. | *No change; high inter-expert variability should be reduced by added guidance. Guidance should mention that details may sometimes be retrieved from tables and figures in the Results.* |
| **Data collection and analysis** | |
| 1. The tests and/or analytical methods used were sufficiently described to allow for evaluation of reliability of results. | *No change; high inter-expert variability should be reduced by added guidance.* |
| 1. The time points for data collection were stated. |  |
| 1. It was stated that the effect of the test compound on cytotoxicity was measured. | *No change; high inter-expert variability should be reduced by added guidance. Guidance to clarify that cytotoxicity is measured as an indication of the viability of the test system.* |
| 1. All results were clearly presented. | *No change; high inter-expert variability should be reduced by added guidance.* |
| 1. The statistical methods and software used were described. | *No change; high inter-expert variability should be reduced by added guidance.* |
| **Funding and competing interests** | |
| 1. The funding sources for the study were stated |  |
| 1. Any competing interests were disclosed or it was explicitly stated that the authors did not have any competing interests. | *No change; high inter-expert variability should be reduced by added guidance. Guidance to clarify expectations so that there is no confusion with e.g., risk of bias analysis.* |
| **Open criterion** | |
|  | *Add open comment criterion based on comments made in the online survey:* Was all information that is indispensable for evaluating the reliability of data given? This includes information on the test compound and controls, test system, study design or study performance. |
