# Supplementary material for: Development of the SciRAP Approach for Evaluating the Reliability and Relevance of in vitro Toxicity Data
Source: Front Toxicol. 2021 Oct 15;3:746430. doi: 10.3389/ftox.2021.746430 (PMC8915875; doi:10.3389/ftox.2021.746430)
Supplement: Supplementary file 3 [file Table9.docx]

Supplementary Material

**Supplementary Table S9**. Changes made to SciRAP *in vitro* methodological quality criteria (version 1.0) based on the expert test round. Criteria marked in blue had high inter-expert variability for one of the 3 studies evaluated by the experts in the test round, criteria marked in green had high variability for 2 or all 3 studies evaluated in the test round. There was pre-existing guidance to support evaluation of the methodological quality in version 1.0, therefore guidance was only improved in version 2.0 where applicable.

| **Version 1.0 – February 2018** | **Adjustments for Version 2.0 – 2021**  *(If nothing stated here no change to v1.0 criterion is made)* |
| --- | --- |
| **Test compound and controls** |  |
| 1. The test compound or mixture was unlikely to contain any impurities that may significantly have affected the results of the study. | *No change, improve guidance to facilitate interpretation of the criterion, including how this criterion can also be used to address interference of the test item itself, such as a nano-material, with the results of the study.* |
| 1. It was likely that the test compound was soluble at the concentrations used. | *No change, improve guidance to facilitate interpretation of the criterion. Also add characterization of phys-chem properties to “open comment criterion”.* |
| 1. An appropriate vehicle was used that is not expected to interfere with the results of the study at the concentration used. | An appropriate solvent (vehicle) was used that is not expected to interfere with the results of the study at the concentration used.  *No other change, improve guidance to facilitate interpretation of the criterion.* |
| 1. An untreated or vehicle control was included. | A solvent (vehicle) control was included.  *This criterion was adjusted based on the high variability in expert ratings as well as expert feedback in the online survey, and based on further cross-checking against terminology in relevant OECD test guidelines.* |
|  | *Add criterion based on comments made in the online survey and also cross-checked against relevant OECD test guidelines:* An appropriate positive control was included, and the expected result was observed from this treatment. |
| **Test system** |  |
| 1. A reliable and sensitive test system (cell line / cells / tissue / organ /embryo) with metabolic competence, if relevant, was used for investigating the test compound and endpoints. | A reliable and sensitive test system (e.g., cell line / cells/ tissue / organ / embryo / sub-cellular fractions) with metabolic competence, if relevant, was used for investigating the test compound and endpoints.  *In addition, improve guidance to facilitate interpretation of the criterion.* |
| 1. Conditions for cultivation and/or maintenance of the cell line / cells / tissue / organ /embryo (incubation temperature, humidity, CO2 concentration, media used, number of cell passages, control of contamination) were appropriate. | Conditions for cultivation and/or maintenance of the cell line / cells / tissue / organ /embryo / sub-cellular fractions (incubation temperature, humidity, CO2 concentration, media used, number of cell passages, control of contamination) were appropriate.  *In addition, improve guidance to facilitate interpretation of the criterion.* |
| **Administration of the test compound** |  |
| 1. The duration of exposure was suitable for the test system and investigated endpoints. |  |
| 1. The concentrations used were suitable for the test system and investigated endpoints. | *Variability between experts in one study is likely due to the characteristics of that study and differences in experience and expertise between experts. Improve guidance to facilitate interpretation of the criterion; make specific reference to available standardized OECD test guidelines and corresponding guidance. The guidance item will also be revised to specifically address considerations of measured concentrations, as well as administered dose.* |
| 1. The test conditions during and after exposure to the test compound were suitable (media and serum used, cell density, incubation temperature, humidity, CO2 concentration). | *Variability between experts in one study is likely due to the characteristics of that study and differences in experience and expertise between experts. Improve guidance to facilitate interpretation of the criterion; make specific reference to available standardized OECD test guidelines and corresponding guidance.* |
| **Data collection and analysis** |  |
| 1. Reliable and sensitive tests and/or analytical methods were used for investigating the endpoints. |  |
| 1. Sufficient numbers of replicates or repetitions of the experiment were used to generate reliable and valid results. | *No change. Update guidance and refer to reference of statistical power calculations and section 8.1 in GIVIMP.* |
| 1. Measurements were collected at suitable time points in order to generate sensitive, valid and reliable data. |  |
| 1. Cytotoxicity was measured and the test compound did not cause cytotoxicity that significantly affected the results. | *No change. Update guidance to clarify how to consider this criterion if the aim of the study is to investigate cytotoxicity.* |
| 1. The statistical methods were clearly described and do not seem inappropriate, unusual or unfamiliar. |  |
| **Open criterion** | |
| 1. Are there any other aspects of study design, performance or reporting that influence reliability? | *Based on comments made in the online survey and review process, add to guidance item as factors that can affect reliability: physicochemical properties of the test compound, knowledge about sensitivity and reliability of commercial kits if used, use of negative control and reference compounds, measurements of final concentrations, blinding of research personnel if relevant (i.e., not automated readout).* |
