# Supplementary material for: Development of the SciRAP Approach for Evaluating the Reliability and Relevance of in vitro Toxicity Data
Source: Front Toxicol. 2021 Oct 15;3:746430. doi: 10.3389/ftox.2021.746430 (PMC8915875; doi:10.3389/ftox.2021.746430)
Supplement: Supplementary file 5 [file Table2.docx]

Supplementary Material

**Supplementary Table S2.** Demographics of participants in the expert round test of SciRAP *in vitro* tool (version 1.0)

| Participants’ demographics | No. of participants in the test round (%) |
| --- | --- |
| Total number of participants | 31* |
| Number of participants that completed the survey | 30 (100) |
| Geographical area |  |
| Europe | 23 (77) |
| USA | 6 (20) |
| South America | 1 (3) |
| Current affiliation |  |
| Academia | 13 (43) |
| Authority and government | 12 (40) |
| Industry and consultancy | 5 (17) |
| Years of experience in conducting in vitro research |  |
| ≤5 | 9 (30) |
| 6–10 | 11(37) |
| >10 | 10 (33) |
| Years of experience in evaluating toxicity studies for health risk assessment |  |
| ≤5 | 15 (50) |
| 6–10 | 7 (23) |
| >10 | 8 (27) |

* 31 participants completed the expert round test, but one participant did not take part to the online survey, where details about participant country of residence, affiliations, and experience were collected.
