# Supplementary material for: Development of the SciRAP Approach for Evaluating the Reliability and Relevance of in vitro Toxicity Data
Source: Front Toxicol. 2021 Oct 15;3:746430. doi: 10.3389/ftox.2021.746430 (PMC8915875; doi:10.3389/ftox.2021.746430)
Supplement: Supplementary file 6 [file Table5.docx]

Supplementary Material

**Supplementary Table S5**. Items to assess relevance of *in vitro* studies (SciRAP tool version 1.0)

| List of proposed relevance items per evaluation domain | |  |
| --- | --- | --- |
| *Test compound* | |  |
| 1. | The identity of the tested substance. | |
| *Test System* | |  |
| 2. | The test system used. | |
| *Endpoint* | |  |
| 3. | The endpoint studied. | |
| *Concentrations* | |  |
| 4. | The concentrations used. | |
|  | |  |
