# Supplementary material for: Development of the SciRAP Approach for Evaluating the Reliability and Relevance of in vitro Toxicity Data
Source: Front Toxicol. 2021 Oct 15;3:746430. doi: 10.3389/ftox.2021.746430 (PMC8915875; doi:10.3389/ftox.2021.746430)
Supplement: Supplementary file 7 [file Table3.docx]

Supplementary Material

**Supplementary Table S3**. Criteria to assess reporting quality (reliability) of *in vitro* studies (tool version 1.0)

| List of proposed criteria per quality domain in the reporting quality subcategory | |
| --- | --- |
| *Test compound and controls* | |
| 1. | The chemical name, ID or CAS-number of the test compound was given. |
| 2. | The purity of the test compound was stated or is traceable according to information given regarding manufacturer and lot/batch number. In case of mixtures, the composition of different constituents was stated. |
| 3. | The solubility of the test compound was described. |
| 4. | The vehicle was described. |
| 5. | It was stated that an untreated or vehicle control was included. |
| *Test System* | |
| 6. | The test system (cell line / cells/ tissue / organ / embryo) was described. |
| 7. | The source of the test system was stated. |
| 8. | Metabolic competence of the test system was described. |
| 9. | The number of cell passages of the cell line used, was stated. (Remove this criterion if the study was not conducted in a cell line.) |
| 10. | Composition of media was described, including use of serum, antibiotics, etc. |
| 11. | Incubation temperature, humidity, and CO2 concentration were described. |
| 12. | Measures taken for avoiding or screening for contamination by mycoplasma, bacteria, fungi and virus were described. |
| *Administration of test compound* | |
| 13. | The administered dose levels or concentrations were stated. |
| 14. | Cell density or number of cells used during treatment was described. (Remove this criterion if the study was not conducted in a cell line.) |
| 15. | The duration of treatment was stated. |
| 16. | The number of replicates per dose level/concentration or the number of times the experiment was repeated was stated. |
| *Data collection and analysis* | |
| 17. | The tests and/or analytical methods used were sufficiently described to allow for evaluation of reliability of results. |
| 18. | The time points for data collection were stated. |
| 19. | It was stated that the effect of the test compound on cytotoxicity was measured. |
| 20. | All results were clearly presented. |
| 21. | The statistical methods and software used were described. |
| *Funding and competing interests* | |
| 22. | The funding sources for the study were stated. |
| 23. | Any competing interests were disclosed or it was explicitly stated that the authors did not have any competing interests. |
