# Supplementary material for: Development of the SciRAP Approach for Evaluating the Reliability and Relevance of in vitro Toxicity Data
Source: Front Toxicol. 2021 Oct 15;3:746430. doi: 10.3389/ftox.2021.746430 (PMC8915875; doi:10.3389/ftox.2021.746430)
Supplement: Supplementary file 8 [file Table4.docx]

Supplementary Material

**Supplementary Table S4**. Criteria to assess methodological quality (reliability) of *in vitro* studies (tool version 1.0)

| List of proposed criteria per quality domain in the methodological quality subcategory | |
| --- | --- |
| *Test compound and controls* | |
| 1. | The test compound or mixture was unlikely to contain any impurities that may significantly have affected the results of the study. |
| 2. | It was likely that the test compound was soluble at the concentrations used. |
| 3. | An appropriate vehicle was used that is not expected to interfere with the results of the study at the concentration used. |
| 4. | An untreated or vehicle control was included. |
| *Test system* | |
| 5. | A reliable and sensitive test system (cell line / cells / tissue / organ /embryo) with metabolic competence, if relevant, was used for investigating the test compound and endpoints. |
| 6. | Conditions for cultivation and/or maintenance of the cell line / cells / tissue / organ /embryo (incubation temperature, humidity, CO2 concentration, media used, number of cell passages, control of contamination) were appropriate. |
| *Administration of test compound* | |
| 7. | The duration of exposure was suitable for the test system and investigated endpoints. |
| 8. | The concentrations used were suitable for the test system and investigated endpoints. |
| 9. | The test conditions during and after exposure to the test compound were suitable (media and serum used, cell density, incubation temperature, humidity, CO2 concentration). |
| *Data collection and analysis* | |
| 10. | Reliable and sensitive tests and/or analytical methods were used for investigating the endpoints. |
| 11. | Sufficient numbers of replicates or repetitions of the experiment were used to generate reliable and valid results. |
| 12. | Measurements were collected at suitable time points in order to generate sensitive, valid and reliable data. |
| 13. | Cytotoxicity was measured and the test compound did not cause cytotoxicity that significantly affected the results. |
| 14. | The statistical methods were clearly described and do not seem inappropriate, unusual or unfamiliar. |
| *Other* | |
| 15. | Are there any other aspects of study design, performance or reporting that influence reliability? |
