# Supplementary material for: Development of the SciRAP Approach for Evaluating the Reliability and Relevance of in vitro Toxicity Data
Source: Front Toxicol. 2021 Oct 15;3:746430. doi: 10.3389/ftox.2021.746430 (PMC8915875; doi:10.3389/ftox.2021.746430)
Supplement: Supplementary file 10 [file DataSheet1.docx]

**Supplementary Figure S1**. Decision tree for evaluating variability in expert ratings for reliability criteria and relevance items, taking into account the distribution of expert evaluations across rating categories, and the percentage of expert ratings for each category. Decision rules are applied to prioritize criteria/items for further evaluation above a pre-defined cut-off for variability.

**
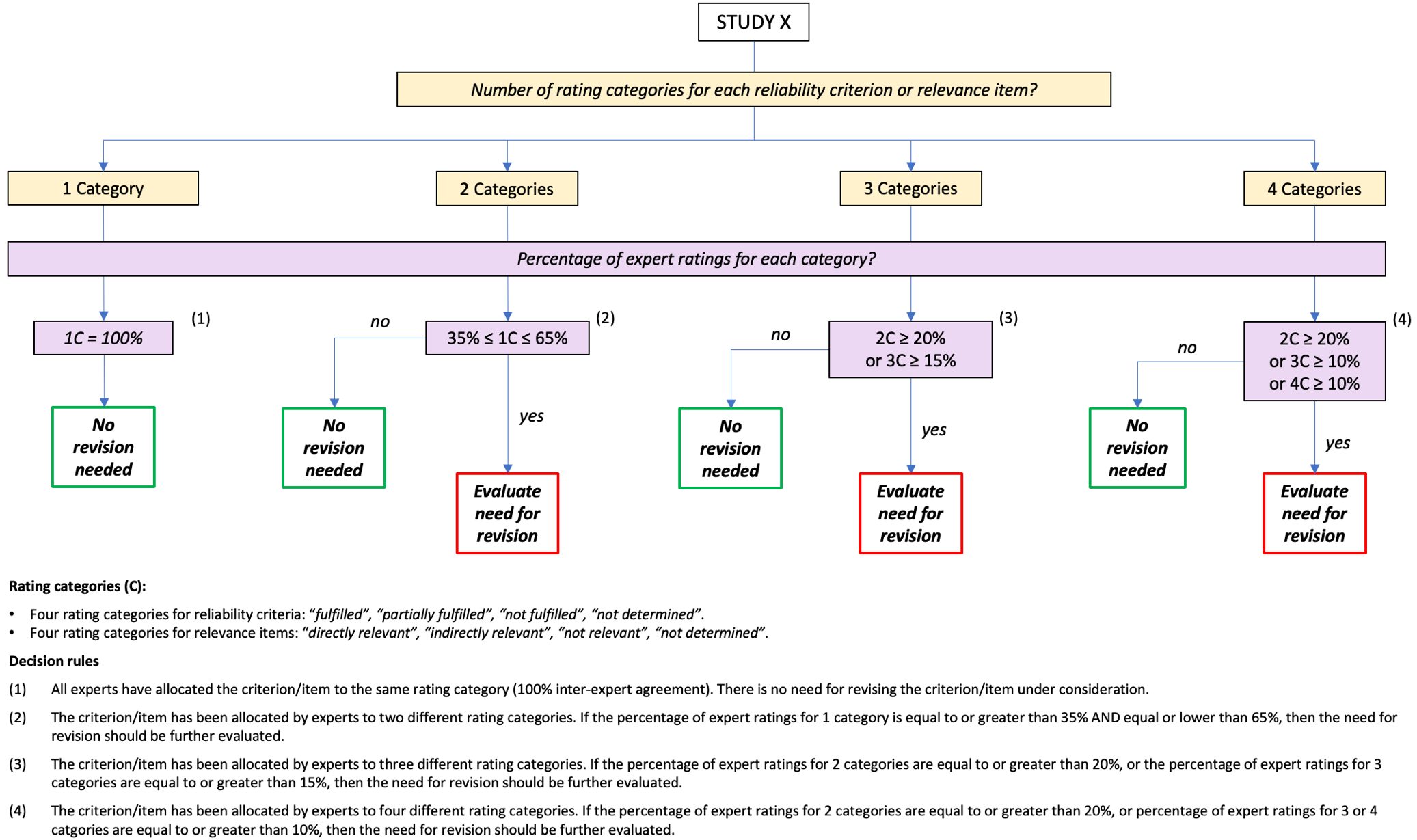
**
